# Dry Matter Intake Prediction from Milk Spectra in Sarda Dairy Sheep

**DOI:** 10.3390/ani13040763

**Published:** 2023-02-20

**Authors:** Antonello Ledda, Silvia Carta, Fabio Correddu, Alberto Cesarani, Alberto Stanislao Atzori, Gianni Battacone, Nicolò Pietro Paolo Macciotta

**Affiliations:** 1 Dipartimento di Agraria, Università di Sassari, 07100 Sassari, Italy; 2 Department of Animal and Dairy Science, University of Georgia, Athens, GA 30602, USA

**Keywords:** multivariate, automatic feeding system, milk spectra

## Abstract

**Simple Summary:**

On livestock farms, measuring individual intake is difficult to assess, but is a very important value for evaluating the feed efficiency of livestock. Feed efficiency has an impact on farm efficiency and, thus, on the economic balance sheet. The purpose of this work was to predict the intake of lactating Sarda ewes from milk spectra using multivariate approaches. Individual intake was found to be moderately correlated with the wavenumbers of the milk spectra. The preliminary results of this study showed that individual intake and, thus, feeding efficiency can be routinely estimated from milk spectra. Further large-scale studies may allow for a rapid, low-cost indicator to be used to keep constant tabs on feeding and farm efficiency.

**Abstract:**

Individual dry matter intake (DMI) is a relevant factor for evaluating feed efficiency in livestock. However, the measurement of this trait on a large scale is difficult and expensive. DMI, as well as other phenotypes, can be predicted from milk spectra. The aim of this work was to predict DMI from the milk spectra of 24 lactating Sarda dairy sheep ewes. Three models (Principal Component Regression, Partial Least Squares Regression, and Stepwise Regression) were iteratively applied to three validation schemes: records, ewes, and days. DMI was moderately correlated with the wavenumbers of the milk spectra: the largest correlations (around ±0.30) were observed at ~1100–1330 cm^−1^ and ~2800–3000 cm^−1^. The average correlations between real and predicted DMI were 0.33 (validation on records), 0.32 (validation on ewes), and 0.23 (validation on days). The results of this preliminary study, even if based on a small number of animals, demonstrate that DMI can be routinely estimated from the milk spectra.

## 1. Introduction

Individual dry matter intake (DMI) is a relevant factor for evaluating feed efficiency (FE) in livestock [1]. In fact, DMI should be carefully considered when formulating animals’ diets, because it is the main driver of animal productivity [2] and because feed represents the main single input cost for the farmers [3]. In the past, most individual DMI measurements were carried out using individual pens, which can increase the anxiety and reduce the performance of the animal [4,5]. This is especially true for sheep, as they are gregarious and social animals [6]. Moreover, the DMI seems to be reduced when social animals (e.g., sheep) are kept in very small groups [7].

Recently, data regarding the individual DMI of animals became available due to the availability of automatic feeding systems at the farm level, which was mainly for experimental purposes [8,9,10,11]. These systems allow not only the individual dry matter intake but also the feeding behavior, to be recorded, e.g., the frequency of access to the manger, the time spent eating, and the feeding routine during the day. However, in this case, routine recording of this trait is hampered by the relatively high costs of these systems [12]. In dairy animals, traits that are difficult to measure directly, such as DMI, can be predicted from spectra obtained from Fourier transform mid-infrared (FT-MIR) spectrometry analysis of milk. Example of variables predicted by milk MIR are fatty acid (FA) profile [13,14,15], methane emissions [16,17], major minerals [18], ration composition [19], energy balance and efficiency [20,21], and dry matter and residual feed intake [22,23]. However, milk spectra have a very large number of variables and, thus, some techniques aimed at reducing the dimensionality of the datasets should be applied. Among the several available approaches, Partial Least Squares Regression (PLSR) and Principal Components Regression (PCR) are probably the most common ones. Even if similar, these two approaches have important differences: the reduction in the number of explanatory variables (i.e., predictors) could be based on variance (PCR) or covariance (PLSR), and it could be carried out either considering (PLSR) or not considering (PCR) the response variable. Both models extract new latent variables (that are combinations of the original predictors), each providing a quota for the total variability of the investigated system. Another technique largely applied to reduce the dimensionality of a dataset is Stepwise Regression (SR), which iteratively selects the predictors most associated with the response variable. Thus, the SR does not modify the original predictors, but only selects the most suitable to predict the investigated trait. 

PLSR has been successfully applied to predict FA profiles [24] and coagulation properties [25] from milk spectra. The prediction of an FA profile from milk spectra allows traditional gas chromatography (GC), which is a time and money consuming process, to be bypassed. This algorithm had a very good coefficient of determination (R^2^), and the GC-measured and PLSR-predicted FA showed similar heritability and prediction accuracy in a genomic study on Sarda dairy sheep [26].

The hypothesis behind this study was that milk spectra, which are now routinely available at the farm level, could be used to predict DMI. This would allow farmers to have a value of DMI for all lactating ewes, even in farms without feeding systems. Thus, the aim of this work was to test the ability of the most widely adopted multivariate techniques to predict DMI from milk spectra in Sarda dairy sheep by using actual DMI values from experimental data.

## 2. Materials and Methods

### 2.1. Data

**Figure 1:**
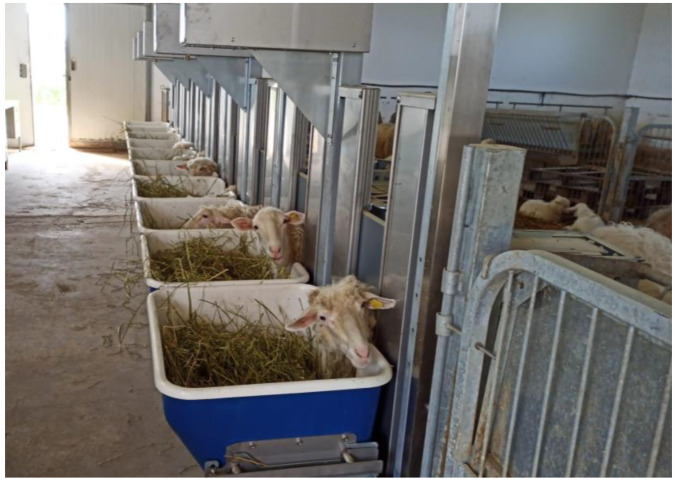
Automatic feeding systems at the experimental farm of the Dipartimento di Agraria, University of Sassari.

A total of twenty-four (24) early-lactation ewes were studied from the experimental flock of the Dipartimento di Agraria, University of Sassari (40°46′24.2″ N, 8°29′31.3″ E; Ottava, SS, 07100, Sassari). The ewes were all pluriparous, and they had an average body weight of 44.38 ± 4.86 kg. After an adaptation period of twenty days, the animals were kept together for twenty-eight days in a barn equipped with ten individual automatic feeding systems (Biocontrol AS, Rakkestad, Norway) and milking equipment (AFIMILK^®^ System, Kibbutz Afikim, Israel). The feeding systems consisted of mangers placed on weighing cells that automatically weighed the feed before and after each animal’s entrance. Access to the feeding systems was controlled by gates that recognized all animals through ear tags and allowed only one animal at a time. Thus, the feeding systems registered the daily DMI for each ewe (Figure 1). Animals were fed a chopped total mix ration (TMR, DM 85.1%; CP 17.6% DM, NDF 34.5% DM, ash 8.3% DM, NEL 1.48 Mcal/kg DM). The TMR contained the following components: polyphyte hay, corn flakes, corn flour, alfalfa hay, soybean flour extract, crushed barley, and molasses.

Animals were milked twice a day, and, during the routine milking, individual milk samples were collected on 8 different days (November–December). Milk samples were corrected according to [27], using 6.5% as fat and 5.8% as protein to obtain FPCM. For each animal, FE was computed as the ratio between the daily milk yield and the dry matter intake. To obtain milk spectra, each milk sample was analyzed by MIR spectroscopy (MilkoScan 6000, Foss Electric). The region spanning from 925.92 and 5011.54 cm^−1^ was considered for the collection of milk spectra. Considering an instrumental resolution of 3.858 cm^−1^, each spectrum consisted of 1060 data points.

### 2.2. Statistical Analysis

Three different techniques were applied to the dataset: PCR, PLSR, and SR. The PCR and PLSR were carried out using the *pcr* and *plsr* functions of the “plsr” R package [28], respectively. For both models, the number of new variables to minimize the root mean square errors of prediction (RMSEP) were selected. SR was carried out using the *step* R function, which selected the variables according to the Akaike Information Criterion (AIC) of the model. In addition, in this case, the best subset of predictors was the one that minimized the AIC.

Three different validation schemes were tested: (i) observations: 90% of observations were randomly assigned to the training dataset, whereas the remaining 10% were used as validation, and this validation scheme was repeated 100 times; (ii) ewes: data from 23 ewes were assigned to the training dataset and used to predict 1 ewe, and this validation was repeated 24 times; and (iii) days: data from 7 ewes were assigned to the training dataset and used to predict 1 day, and this was repeated 8 times. The prediction ability of each model was evaluated by the correlation between the measured real dry matter intake (rDMI) and the estimated DMI (eDMI) from the spectra, averaged across replicates.

The three models and the three validation schemes were tested using the raw spectra or the spectra without the wavenumbers associated with water.

## 3. Results

The mean daily FPCM across the whole experiment was 1.56 ± 0.39 kg/day, and it ranged from 0.75 to 2.49 kg/day. According to the median FPCM, animals were divided in two classes: high and low MY. The average FPCM values in the two classes were 1.25 ± 0.17 kg/day and 1.88 ± 0.26 kg/day for low-yielding and high-yielding ewes, respectively. These values were associated with three different DMIs, measured on the same day and one and two days before the milking event.

The average individual DMI registered the same day of the milking event in the experimental group was 2.00 ± 0.63 kg/day (Table 1), and ranged from 0.3 to 4.5 kg. When considering the DMI measured on the same day as the milking event, high-yielding ewes showed a DMI equal to 2.07 ± 0.68 kg, whereas the low-yielding ewes had a DMI of 1.94 ± 0.58 kg. The same pattern was observed one and two days before the milking event (Table 1).

**Figure 2:**
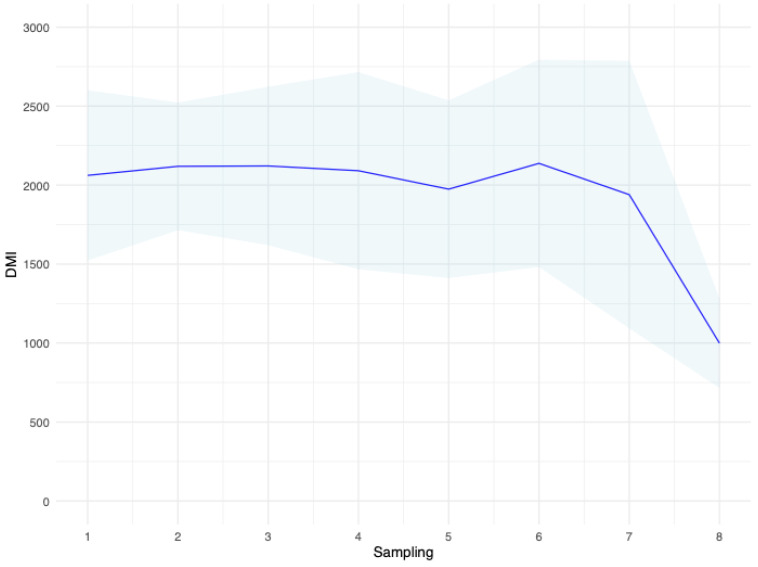
Average dry matter intake (blue line) and its standard deviation (light-blue region) across the eight samplings.

The overall average FE (considering the DMI measured the same day of the milking event) was 0.87 ± 0.41 kg of FCPM/kg of dry matter intake, and it ranged from 0.3 to 2.5. When computed individually for each sheep, the FE (on the day of milking) ranged from 0.48 to 1.38 kg of FCPM per kg of dry matter intake. The FE was almost stable across the eight sampling points, with only the last day showing a larger value due to a general decrease in the dry matter intake (Figure 2). High-yielding ewes showed greater FE compared to the low-yielding ewes (Table 1).

In this study, the correlations between DMI and FPCM were not significant considering the pooled records of all the ewes (Table 2).

**Figure 3:**
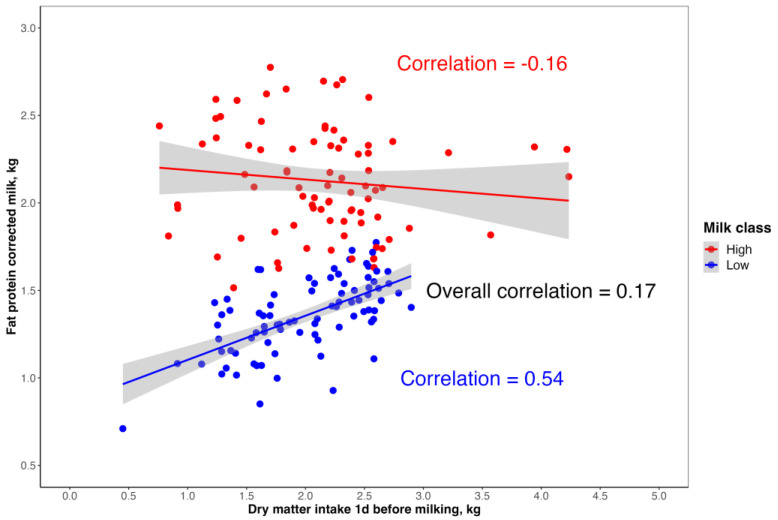
Relationship between fat- and protein-corrected milk (FPCM) and dry matter intake measured the day before milking. Red indicates high-yielding ewes, whereas blue indicates low-yielding ewes. The lines indicate the trend line from the linear model.

However, low-yielding ewes showed positive correlations with DMI that ranged from 0.34 to 0.54, whereas high-yielding ewes showed negative correlations (Table 2 and Figure 3).

The correlations between DMI and FPCM were computed for each sampling day and animal. Correlations between the daily DMI (measured on the day of the milking event) and the daily FPCM within each sampling point ranged from −0.11 (at the fourth sampling day) to 0.43 (computed using data from the eighth sampling day). The correlations between DMI and FPCM were also computed for each sheep: these values ranged from −0.74 to 0.77. Correlations considering the DMI measured one and two days before the milking events showed a similar pattern.

The significant variability among the animals could also be observed while computing the correlations between FPCM and the DMI measured the day before the milking event (Figure 3). However, when the animals were considered all together, a correlation of 0.17 (Table 2 and Figure 3) was observed for the DMI one day before of the milking event (Table 2); thus, this value was used in the prediction models.

Table 3 shows the results of the individual DMI prediction (measured one day before the milking event) based on the milk spectra. On average, PCR and PLSR selected 11 and 2 new variables from the raw spectra, and 18 and 7 new variables from the spectra without the wavenumbers associated with water. SR showed the opposite behavior, i.e., it selected more variables when applied to the raw spectra than to the spectra without the wavenumbers associated with water. On average, SR selected 81 and 54 wavenumbers from the spectra with or without water, respectively.

Correlations between rDMI and eDMI were low to moderate, and ranged from 0.16 (PLSR applied to predict DMI of ewes on the spectra without wavenumbers associated with water) to 0.38 (PLSR applied to predict DMI of observations and ewes on the raw spectra). Standard errors for the correlations ranged from 0.02 to 0.11. The average correlation between rDMI and eDMI for the three validation schemes (across models and spectra) were 0.33, 0.32, and 0.23 for observations, ewes, and days, respectively (Table 3).

**Figure 4:**
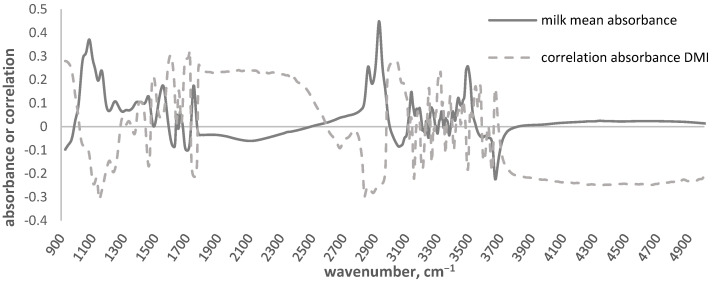
Plot of the overlapping values of the correlation between the dry matter intake (DMI) and the milk absorbance at given wavenumber, and that of the average MIR spectra of milk samples.

Figure 4 illustrates the correlations between DMI and milk absorbance at each wavenumber. The average correlation between DMI and all wavenumbers was −0.03 ± 0.19, and ranged from −0.31 to 0.32.

The regions showing the highest correlations between the DMI and milk absorbance were between ~1100 and 1300 cm^−1^ and between ~2800–3000 cm^−1^. However, as reported in Figure 3, some other regions showed moderate correlations and included wavenumbers which had often been discharged due to the water absorption, as well as others belonging to a large area (~1770–2400 cm^−1^).

## 4. Discussion

Individual DMI is an important factor that could provide information regarding the FE of animals. Its recording on a large scale is difficult because dairy ewes are traditionally fed all together. Recently, the automatic feeding systems that can record individual DMIs have been spreading in sheep farms. However, the availability of these systems is still quite low because of their high cost. Thus, the prediction of individual DMI could help to save money and could provide important information at the population level. Besides the use of milk spectra, several mathematical methods to empirically estimate dry matter intake have previously been developed for small ruminants, and have also been applied to Sarda dairy sheep [27]. The main variables driving DMI are metabolic body weight (i.e., body weight, kg^0.75^) and production of fat- and protein-corrected milk (FPCM; 6.5% fat and 5.8% protein) as follows: DMI, kg/d per ewe = −0.545 + 0.096 × metabolic weight + 0.650 × FPCM [27]. This equation was applied to the average values of body weight and FPCM recorded in the present study, and it estimated a DMI of 2.10 kg/day, which is very close to the values recorded by the automatic feeding systems in this trial (Table 1). Moreover, the average values of DMI and MY found in this study are in line with previous studies on lactating Sarda dairy sheep. Cabiddu et al. [29] analyzed the dry matter intake of ewes during the late pregnancy and suckling periods, and they reported an average value of 1.71 kg per day (from 1.58 to 1.87 kg per day according to the diet, with an average milk yield of 1.58 ± 0.10 L/day). A slightly lower average value (1.3 kg/day) was reported by Lunesu et al. [30], who investigated the effects of dietary starch concentration on the performance of mid-lactating ewes. The DMI reported by these authors ranged from 1.2 to 1.4 kg per day according to the diet, with an average MY of 0.9 kg/day [30]. Finally, Carta et al. [31] found an average dry matter intake of 1.8 kg/day in Sarda ewes, with an average MY of 1.3 kg/d. Muir et al. [32] analyzed the DMI in composite sheep, and they reported an average value of 2.7 ± 0.42 kg per day in adult ewes. This composite breed had average weight of 62.0 ± 6.8; thus, the greater DMI values could be due to the greater average body weight. The average DMI in New Zealand sheep was reported to be 0.92 kg/d (ranging from 0.45 and 1.55 kg/d, depending on the experiment and the diet [33]).

As reported in Table 2, correlations between DMI and FPCM varied according to the animals considered: the correlations were positive when computed on low-yielding ewes and negative when considering high-yielding animals. This result suggests that the relationship between DMI and milk production was affected by the feed efficiency of the animals and the depletion of body reserves. Moreover, the association between DMI and MY should be evaluated, also considering the body condition scores (BCS) and body weight changes. In order to better decipher the relationship between the two parameters, the correlations between DMI and FPCM were computed for each individual sampling day and animal. Greater variability was observed when correlations between DMI and FPCM were computed for each sheep. These results highlight a huge level of variability due to environmental (sampling day) and genetic (animal) factors that influence the dry matter intake in sheep, as pointed out by the analysis of the FE reported in Table 1. Moreover, the results regarding the correlations between DMI and FPCM could be due to the fact that, even after a long adaptation period, some animals were not well adapted to the use of the automatic feeding systems. The low correlation estimated between pooled DMI and FPCM data seems to disagree with the strong relationship between milk production and energy supply, for which an increased DMI has been considered the main driver [27,34]. Stronger correlations between DMI and MY were reported in cattle: from 0.52 to 0.85, according to the breed, parity, and feeding system [35,36]. However, Liang et al. [37] analyzed the total DMI or the DMI within two hours of the feed suppletion, and they estimated correlations with DMIs of 0.38 and −0.02, respectively. Zamuner et al. [38] reported a Spearman rho correlation of 0.70 between DMI, as a percentage of body weight, and fat- and protein-corrected milk in dairy goats from 14 to 42 days in milk. The greater average correlations computed between DMI and MY in dairy cattle could be explained by the stronger genetic correlation of this species, which leads to more efficient cows which are more similar to each other compared to the ewes studied herein.

In the present study, when all ewes were considered together, the strongest correlation was found between MY and DMI one day before the milking event (Table 2). This result could be due to the fact that milk released in the morning of one day was accumulated in the interval between the previous and the subsequent milking and, thus, the milk yield was affected by the amount and composition of the feed eaten by the animals the day before. For this reason, the DMI measured the day before of the milking event was used as the dependent variable in the models to predict this parameter from the milk spectra. The lower number of new variables explaining the total variability found when PCR and PLSR were applied to the raw spectra could be associated with the low variability of wavenumbers associated with water (i.e., 1582–1701 and 3048–5000). On the contrary, the SR selected more wavenumbers when applied to the raw spectra, since the latter had more input data compared to the spectra without the wavenumbers associated with water.

Looking at the prediction ability (i.e., correlations between rDMI and eDMI) of the three models involved, PLSR showed better performance on the raw spectra, whereas PCR worked better using the spectra without the wavenumbers associated with water. SR was the best model to predict the DMI on a particular day using both spectra, with or without the wavenumbers associated with water. It should be pointed out that SR has the advantage of selecting the wavenumbers without extracting new variables, as both PCR and PLSR do. Therefore, once a subset of wavenumbers is selected, these can be easily propagated to the whole population to better predict DMI. Moreover, the selection of wavenumbers instead of new predictors (i.e., principal components) allows for a biological interpretation of the results. Moreover, the results of the prediction analysis (i.e., low-moderate correlations between real and estimated DMI) could be partially due to the weak correlation computed between DMI and milk production in the present study, since the milk spectra reflect the milk production.

The lower predictive ability of the models applied to predict DMI on a particular day could be ascribed to the larger between-day variability, especially on the last sampling day (as observed in Figure 2). The exclusion of one animal at the time has already been used as a validation scheme in the prediction of DMI from milk spectra [12,22]. Shetty et al. [22] applied PLSR to predict DMI from milk spectra in dairy cows, and the correlations between rDMI and eDMI ranged from 0.49 to 0.55. Recently, PLSR has been applied to milk infrared spectra to predict DMI in 552 Dutch Holstein cows; a correlation of 0.47 between rDMI and eDMI was estimated [39]. Dórea et al. [12] predicted the DMI of 308 mid-lactating dairy cows, testing different models. When these authors used PLSR on the whole or restricted milk spectra only, they reported correlations between rDMI and eDMI of 0.17 and 0.36, respectively. The generally larger predictive abilities of the models found in these studies could be ascribed to the larger dataset and the different species. Cows, and especially some dairy breeds such as Holstein, have been strongly selected for milk yield and, therefore, for DMI, as demonstrated by the very strong correlation reported in this species between these two traits. Thus, the DMI is more stable across cows than ewes.

The useful potential of milk MIR spectroscopy as a phenotyping tool has already been widely studied; this technique is routinely used to determine the main components of milk (fat, protein, and lactose) in the framework of milk payment and milk recording, but also other minor compounds [40,41]. Moreover, recently, several studies have been conducted to predict more complex phenotypes, such as the milk fatty acid profile, cheese-making aptitude, metabolic status of animals, feed composition, body weight, methane emissions, and feed intake [41].

As shown in Figure 4, the overlapping with the mean absorbance of milk spectra allows to identify the region and the wavelengths more associated with the DMI. The regions showing the highest correlations were between ~1100 and 1300 cm^−1^ and between ~2800–3000 cm^−1^. The first MIR spectral region includes signals related to milk lactose content (from ~1100 to 1200), due to the presence of C-O bonds belonging to the alcoholic and ether groups of carbohydrates [42,43], and to the protein content (from ~1200 to 1300 cm^−1^), due to the presence of C-N bonds of tertiary amides [43]. The second MIR spectral region includes signals related to the asymmetric and symmetric stretching of methylenic C–H fat bond [44]. The importance of fat, protein, and lactose regions of the milk infrared spectral profile in relationship to the DMI was consistent with a previous work on dairy cows [22]. However, as it can be seen in the Figure 3, other regions showed not negligible correlations; some of these include wavelengths often discharged due to the water absorption, and others belong to a large area that is not typically related with milk components (~1770–2400 cm^−1^) but were found to be important for other milk traits, as milk coagulation properties [45,46]. This finding disagrees with Shetty et al. [22] which affirmed that milk spectral data does not add significant new information to improve DMI prediction models.

This study investigated the relationship between milk production and individual DMI in lactating sheep and the possibility of using milk spectra to predict the latter. The increase in knowledge on this field (i.e., precision farming) could lead to several advantages to the livestock sector in terms of saving time, managing animals, and reducing waste. The results regarding the association between MY and DMI could have a strong impact on the management of dairy farms. In fact, since these two traits had different correlations in low- and high-yielding ewes, the farmers might divide the flock into groups according to the different levels of milk production, thus increasing the farm’s efficiency through better feed utilization and a decrease in waste. Waste reduction and a better utilization of feed in the farms allow a decrease in the production costs and an increase in the economic income of the farmers. Moreover, good management of the animals has a positive effect on the environmental impact of farms, because DMI is a crucial parameter to define livestock efficiency. The animal production system is called to meet the demand of consumers who want to reduce the environmental impact of the livestock sector without a decrease in production levels. The DMI predictions could be a great way to increase the productivity of the farms without any additional costs. The results of the present study suggest the possibility of predicting DMI from milk spectra, allowing farmers to have records for all lactating ewes, even in farms without the automatic feeding systems. The suggested methodology needs to be improved and tested on a larger dataset with more observations in order to increase the predictions’ accuracy.

## 5. Conclusions

This is a preliminary study based on a small number of observations that, despite the low to moderate correlations between real and estimated dry matter intake, demonstrated that these prediction models can be used to routinely predict the DMI of animals from milk spectra. Future studies on a larger dataset that also consider BCS and body weight changes might be needed. The PLSR gave the best results, both with raw spectra and without water variables; thus, it should be particularly considered for further applications. The inclusion of more observations in the future, especially from animals in different stages of lactation and with different diets, is expected to increase the prediction ability.

## Figures and Tables

**Table 1:** Individual fat–protein-corrected milk (FPCM), dry matter intake, and feed efficiency during the experimental trial.

	FPCM Class		
Trait	All	Low	High
**FPCM yield**	1.56 ± 0.39	1.25 ± 0.17	1.88 ± 0.26
**Dry matter intake**			
Day of milking	2.00 ± 0.63	1.94 ± 0.58	2.07 ± 0.68
1 d before milking	1.93 ± 0.67	1.88 ± 0.57	1.98 ± 0.76
2 d before milking	2.07 ± 0.62	2.00 ± 0.55	2.15 ± 0.68
**Feed efficiency**			
Day of milking	0.87 ± 0.41	0.71 ± 0.28	1.04 ± 0.46
1 d before milking	0.96 ± 0.61	0.73 ± 0.29	1.18 ± 0.76
2 d before milking	0.84 ± 0.43	0.67 ± 0.24	1.01 ± 0.51

FPCM = fat- and protein-corrected milk.

**Table 2:** Correlations between fat- and protein-corrected milk (FPCM) and dry matter intake measured either on the day of or one or two days before the milking event.

	Dry Matter Intake		
FPCM Class	Same Day	1 d Before	2 d Before
All	0.11 ^NS^	0.17 ^NS^	0.06 ^NS^
Low	0.40 ***	0.54 ***	0.34 **
High	−0.14 ^NS^	−0.16 ^NS^	−0.27 *

^NS^ = not significant; * = *p* < 0.05; ** = *p* < 0.01; *** = *p* < 0.001. FPCM = fat- and protein-corrected milk.

**Table 3:** Results of the dry matter intake prediction and average correlations (standard errors) between real and estimated dry matter intake.

	Raw Spectra			Without Water			
Validation	PCR ^1^	PLSR ^2^	SR ^3^	PCR	PLSR	SR	
	Number of variables						
Observations	11 (1)	2 (0)	88 (7)	18 (1)	6 (0)	61 (7)	
Ewes	11 (2)	2 (0)	79 (15)	17 (0)	7 (0)	54 (14)	
Day	11 (3)	3 (0)	76 (27)	18 (3)	8 (2)	47 (19)	
	**Correlation between real and estimated dry matter intake**						**Average**
Observations	0.26 (0.02)	0.38 (0.02)	0.31 (0.02)	0.36 (0.02)	0.33 (0.02)	0.33 (0.02)	0.33
Ewes	0.29 (0.06)	0.38 (0.06)	0.30 (0.07)	0.35 (0.06)	0.29 (0.05)	0.32 (0.06)	0.32
Day	0.22 (0.06)	0.28 (0.05)	0.29 (0.11)	0.20 (0.06)	0.16 (0.05)	0.23 (0.07)	0.23

^1^ PCR = principal components regression; ^2^ PLSR = partial least squares regression; ^3^ SR = stepwise regression.

## Data Availability

The data presented in this study are not publicly available.
